# CFD modeling of incinerator to increase PCBs removal from outlet gas

**DOI:** 10.1186/s40201-015-0212-0

**Published:** 2015-08-12

**Authors:** Kamyar Yaghmaeian, Nematallah Jaafarzadeh, Ramin Nabizadeh, Golbarg Dastforoushan, Jalil Jaafari

**Affiliations:** Department of Environmental Health, School of Public Health, Tehran University of Medical Sciences, Tehran, Iran; Center for Solid Waste Research, Institute for Environmental Research, Tehran University of Medical Sciences, Tehran, Iran; Environmental Technologies Research Center, Ahvaz Jundishapur University of Medical Sciences, Ahvaz, Iran; Department of Environmental Engineering, Islamic Azad University, Tehran, Iran; Department of Environmental Health, School of Public Health, Guilan University of Medical Sciences, Rasht, Iran

**Keywords:** Incinerator, PCB, Fluent model, Baffle, Thermal degradation

## Abstract

Incineration of persistent organic pollutants (POPs) is an important alternative way for disposal of this type of hazardous waste. PCBs are very stable compounds and do not decompose readily. Individuals can be exposed to PCBs through several ways and damaged by their effects. A well design of a waste incinerator will convert these components to unharmfull materials. In this paper we have studied the design parameters of an incinerator with numerical approaches. The CFD software Fluent 6.3 is used for modelling of an incinerator. The effects of several baffles inside the incinerator on flow distribution and heat is investigated. The results show that baffles can reduce eddy flows, increase retaining times, and efficiencies. The baffles reduced cool areas and increased efficiencies of heat as maximum temperature in two and three baffle embedded incinerator were 100 and 200 °C higher than the non-baffle case, respectively. Also the gas emission leaves the incinerator with a lower speed across a longer path and the turbulent flow in the incinerator is stronger.

## Introduction

Waste incineration is a well-established treatment technology for municipal, industrial, hospital and hazardous wastes [1–3]. It is also one of the most frequently selected method of waste management for no-longer reusable or recyclable industrial products and materials [4–8]. In the some part of waste incinerators, persistent organic pollutants (POPs) are formed due to the presence of products of incomplete combustion, oxygen and chlorine at temperatures between 200 and 800 °C [9, 10]. The final solution for persistent organic pollutants (POPs) such as polychlorinated biphenyls (PCBs) that cannot be recycled or landfilled is to use an incinerator [11]. The minimum residence time suggested for removal of PCBs in an incinerator is about 2 s at 1200 °C or 1.5 s at 1600 °C [11]. This can be achieved only through increasing the residence time or improved heat distribution. Measurements indicate that most PCBs incinerators are not able to provide these conditions due to the presence of inefficient cold zone with low efficiency in terms of mixing and heat distribution [12, 13]. Furthermore, in the cold zone of waste incinerators polychlorinated biphenyls (PCBs) are formed [10, 14–16].

In recent years, many attempts have been made to model processes in incinerators. San José et al., investigated the effect of incinerator efficiency on the emissions in an industrial area with the help of MM5, CMAQ and EMIMO [17]. The results showed that the effect of emissions from incinerator is insignificant compared to the surrounding industries and highways. The effect was comparable just in the case of ozone. Stanmore et al., modeled the formation of PCDD/F in municipal and hospital incinerators and proposed a general empirical model to calculate the level of gaseous and solid PCDD/F [18].

In another study, Goh et al., modeled the combustion bed of a municipal incinerator and proposed a comprehensive flexible model [19]. The results of the model were used as boundary conditions for modeling upper gases in CFD models. The model results can also be used to optimize the incinerator and reduce the production of waste sludge and waste mixing [19]. In a similar study, the waste mixing was modeled before burning in an incinerator. In this study, a mathematical model was proposed for simulating waste mixing in the incinerator and the model results were compared with experimental results. Huang, used a kinetic model of reaction to model the formation PCDD/F in an industrial incinerator [20]. The model variables include the formation and removal rates of PCDD/F, carbon gasification, partial pressure of oxygen and equations for temperature and time. A good agreement was obtained between the experimental and model results. Khiari et al., proposed a mathematical model for dynamic simulation of an incinerator [21]. The lower part of the incinerator and waste pyrolysis were modeled. The model results were compared with the results of similar studies. Thomas offered an one-dimensional model to simulate the incineration of emissions in an incinerator [22]. Taking into account radiation, convection and conduction heat transfer processes, and the gas flow was simulated. The heat capacity of gases, thermal conductivity and viscosity effects were included considering the temperature dependence of the reaction.

The main objective of this study was to investigate the explores ways to optimize the efficiency of the PCBs removal in incinerators in the presence of baffles embedded in the combustion chamber. Modeling was performed first with 2 and then with 3 baffles.

## Materials and methods

### Incinerator specification

A rotational PCBs incinerator with one inlet and one outlet for pollutants was studied. The incinerator was designed for one of PCBs isomers called Arochlorine-1242 (a mixture of 1 % C_12_H_9_Cl, 13 % C_12_H_8_Cl_2_, 45 % C_12_H_7_C_l3_, 31 % C_12_H_6_C_l4_ and 10 % C_12_H_5_Cl_5_ with average Cl content of 42 %. Arochlorine-1242 is among the most commonly used types of PCBs, especially in power transformers in great plants. The specification of the incinerator was selected according to Theodore and Reynolds [23]. The Arochlorine-1242 incinerator was designed manually and with the help of software. The design principles in different parts of incinerator such as primary and secondary combustion chambers, furnaces, boilers, suppressor devices and air pollution control devices were similar to the incinerator modeled in [23]. Design calculations were performed according to Charles’s law and Dulong’s equation. Table 1 summarizes the specification calculated for the incinerator. The technical specifications presented in Table 1 were used as initial inputs to Fluent model for simulating the incinerator.

**Table 1:** The technical specification of the incinerator

Value	Parameter
2270 (kg/h)	Maximum capacity
C_12_H_7_Cl_3_	Input pollutant
20,277 (KJ/Kg)	Net thermal value
50 %	Excess air
2.76 m	Initial diameter
11 m	Initial height
0.5 m	Inlet and outlet diameter
2.7 s	Initial residence time

### The equations governing the pollutant flow in the incinerator

Given the air flow velocity and the dimensions of the incinerator as well as the high temperatures, the flow regime is turbulent. Neglecting the net rotating flows, since all changes along the flow and in vertical direction are important, the k-ε turbulence model is a good model for analyzing this problem. The equations required to solve the isothermal gas flow in the incinerator include time-averaged mass and momentum conservation equations [24]:

Mass conservation $$ \frac{\partial {U}_i}{\partial {X}_i}=0 $$ (1) 

Momentum conservation

2 $$ \frac{\partial \rho {U}_i}{\partial t}+\frac{\partial \rho {U}_i{U}_j}{\partial {X}_i}=\frac{\partial p}{\partial {X}_i}+\rho \frac{\partial }{\partial {X}_j}\left[v\left(\frac{\partial {U}_i}{\partial {X}_i}+\frac{\partial {U}_i}{\partial {X}_i}\right)\right]-\frac{\partial {U_i}^{\prime }{U_i}^{\prime }}{\partial {X}_j}+{S}_{Mi} $$

Where U_i_ is velocity along i, i = 1, 2, 3, X_i_ is x, y, z coordinates along i, Y mass fraction of gas emissions, ρ air density, υ kinematic viscosity, U_i_ turbulent velocity component along i’ and S_Mi_ is the momentum source along i’.As mentioned previously, since the Reynolds removal process and time-averaged equations will lead to unknown relationships for fluctuating velocity components, so a turbulent model is also needed. Thus, the k-ε model was used. This model requires the solution of two additional transport equations, one for turbulent kinetic energy, k and the other for its dissipation rate or ε [24]:

3 $$ \frac{\partial }{\partial {x}_i}\left(\rho {u}_ik\right)=\frac{\partial }{\partial {x}_i}\left[\left(\frac{\mu +{\mu}_t}{\delta_k}\right)\left(\frac{\partial k}{\partial {x}_i}\right)\right]+P-\rho \varepsilon $$

4 $$ \frac{\partial }{\partial {x}_i}\left(\rho {u}_i\varepsilon \right)=\frac{\partial }{\partial {x}_i}\left[\left(\frac{\mu +{\mu}_t}{\delta_{\varepsilon }}\right)\left(\frac{\partial \varepsilon }{\partial {x}_i}\right)\right]+{C}_1\frac{\varepsilon }{k}P-{C}_2\rho \frac{\varepsilon^2}{k} $$

Enthalpy conservation: $$ \frac{\partial }{\partial {x}_i}\left(\rho {u}_ih\right)=\frac{\partial }{\partial {x}_i}\left[\left(\frac{\mu +{\mu}_t}{\delta_h}\right)\left(\frac{\partial h}{\partial {x}_i}\right)\right]+{S}_h $$ (5)

Chemical species conservation: $\frac{\partial }{\partial {x}_i}\left(\rho {u}_i{m}_s\right)=\frac{\partial }{\partial {x}_i}\left[\left(\frac{\mu +{\mu}_t}{\delta_h}\right)\left(\frac{\partial {m}_s}{\partial {x}_i}\right)\right]+{S}_s$ (6)

Equation of State: $\rho =\frac{P}{RT\sigma {m}_j/{M}_j}$ (7)

### Incinerator simulation

**Fig. 1:**
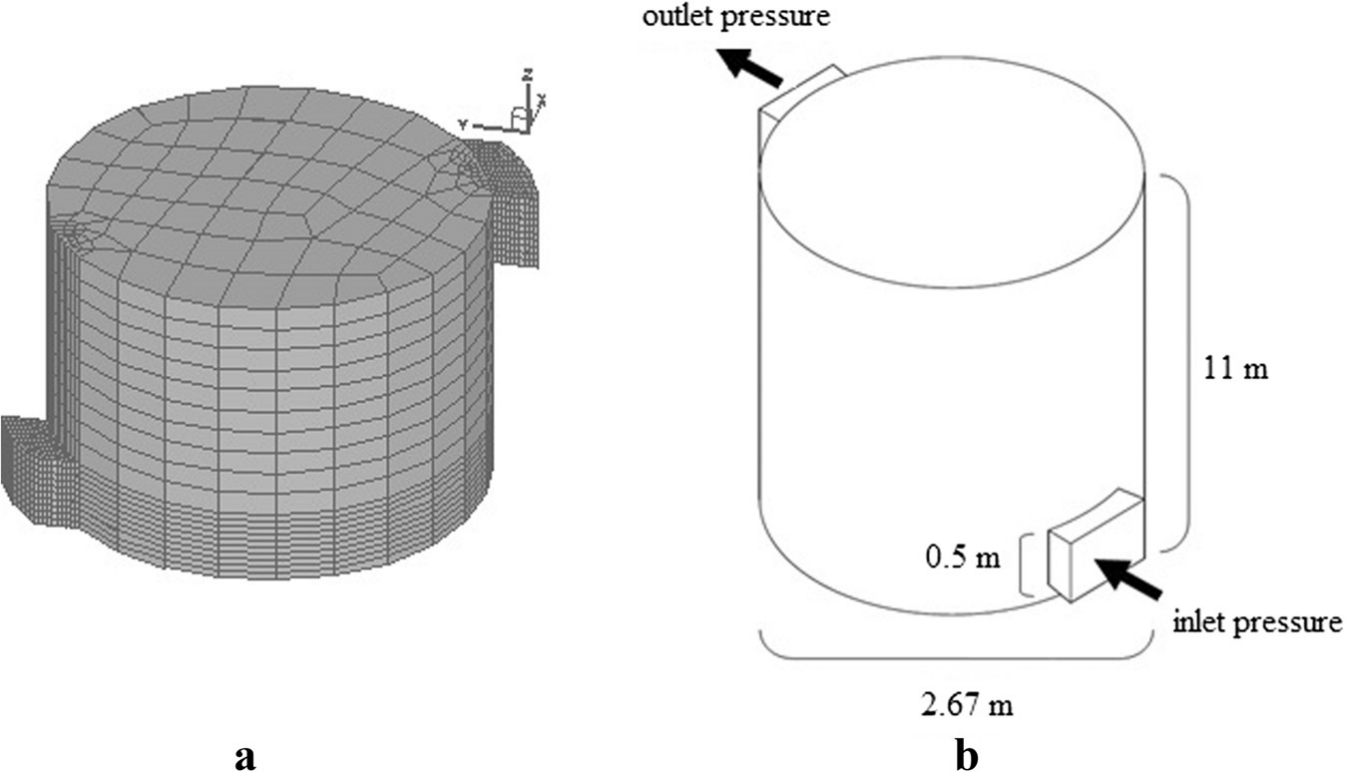
**a** A view of the meshed model of the incinerator, (**b**) The geometrical charactersitics of the incinerator and boundary conditions

To simulate the studied incinerator, a Cartesian coordinate system with the dimensions of 20 × 20 × 20 mm containing 10,424 control volumes was used. The x, y and z axes represent the length, width and height, respectively [24]. In the regions where sharp gradients in the variables are expected, fine meshing is considered as possible. Structured meshes were used for meshing the model, because the number and distribution of meshes in different solution regions can be controlled. In addition, the boundary conditions can be well defined [24]. The boundary layer mesh generator was used in the regions where thermal analysis was important. Fig. 1a and b show the model, the meshed model and boundary conditions.

Wall boundary condition was considered on the studied incinerator, because the solid surface is in direct contact with the fluid [24]. Given that the size and profile of the inlet pressure to the studied incinerator are known, the pressure inflow and pressure outflow were selected for inlet and outlet boundary conditions, respectively. The walls were made of steel with a thermal conductivity of 202 W/M.K. For the flows with a velocity less than the speed of sound, a turbulence of less than 5 % can be considered as assumed in the present model. Other inputs are described in Table 2.

**Table 2:** The inputs to the Fluent model

Value	Parameter
3 %	Turbulent Intensity
Stationary	Wall Motion
No slip	Shear Condition
0.00004 (m)	Roughness Height
2.76 (m)	Hydraulic Diameter
0.5	Roughness Constant

## Results and discussion

**Fig. 2:**
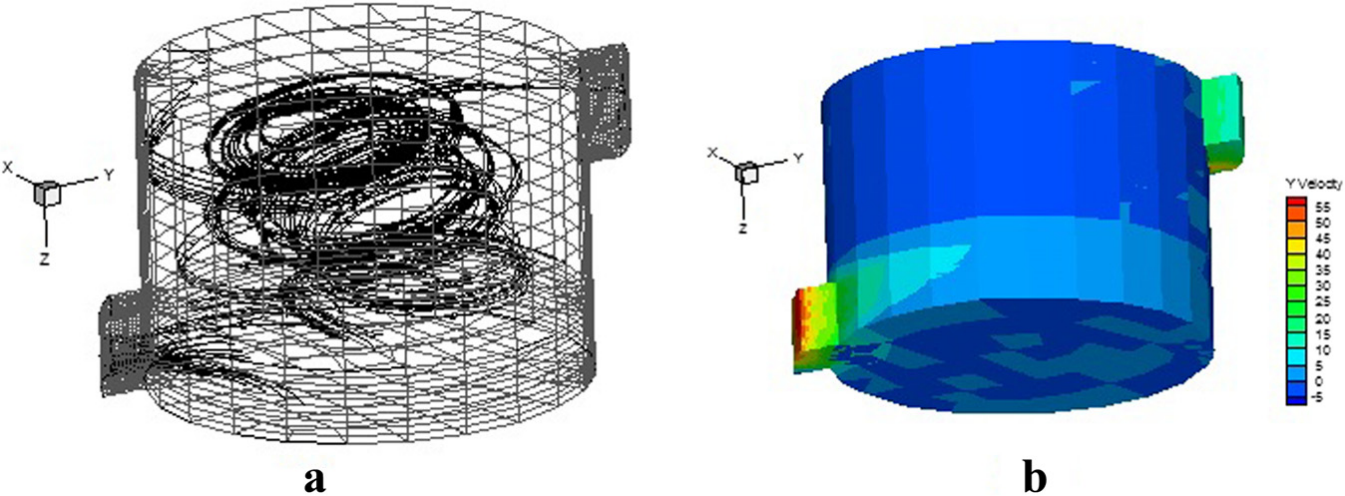
**a** The motion path of gases in the incinerator in normal conditions, (**b**) Iso-velocity contours in the incinerator in normal conditions

Figure 2a shows the motion path of emissions in the studied incinerator. As expected, due to the lack of baffles, incinerator geometry as well as the tangential flow of emissions, large rotating bubbles are formed in the incinerator. Rotating regions (eddies) are formed when the flow pressure reduces the kinetic energy of the fluid particles causing stagnant points in the flow. In addition, there is also a secondary flow that directs the flow downward [25]. Fig. 2b shows the iso-velocity contours in the initial state (without baffles). As seen, increasing the inlet gas temperature will increase the velocity (compared to the isothermal case). A maximum velocity of 55 m/s occurs in the incinerator outlet. In this case, the high speed gas particles quickly leave the incinerator causing a decrease in the residence time. In this case, the average residence time of gaseous emissions is 3 s.

**Fig. 3:**
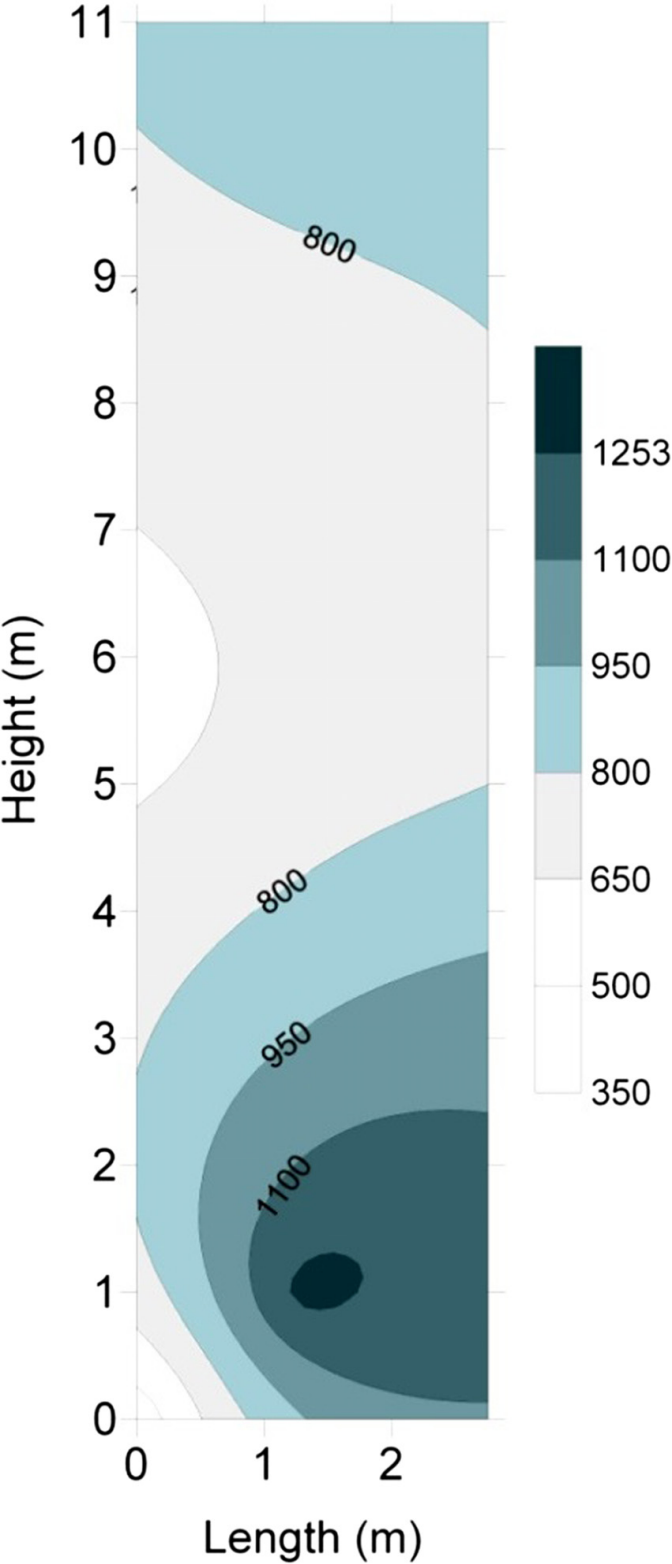
The temperature profile (°C) inside the incinerator in normal condition

Figure 3 shows the predicted temperatures. Temperature increases after the gaseous emissions entering the lower part of the studied incinerator. The temperature inside the studied incinerator reaches over 1300 °C. The predicted temperatures demonstrate cold zones in the incinerator where air is mixed with gas eddies and thus temperature decreases to 600 °C. The temperature drop in the incinerator may significantly affect the composition of the exhaust gases from the incinerator preventing the removal of PCB bonds.

### Efficiency Optimization

**Fig. 4:**
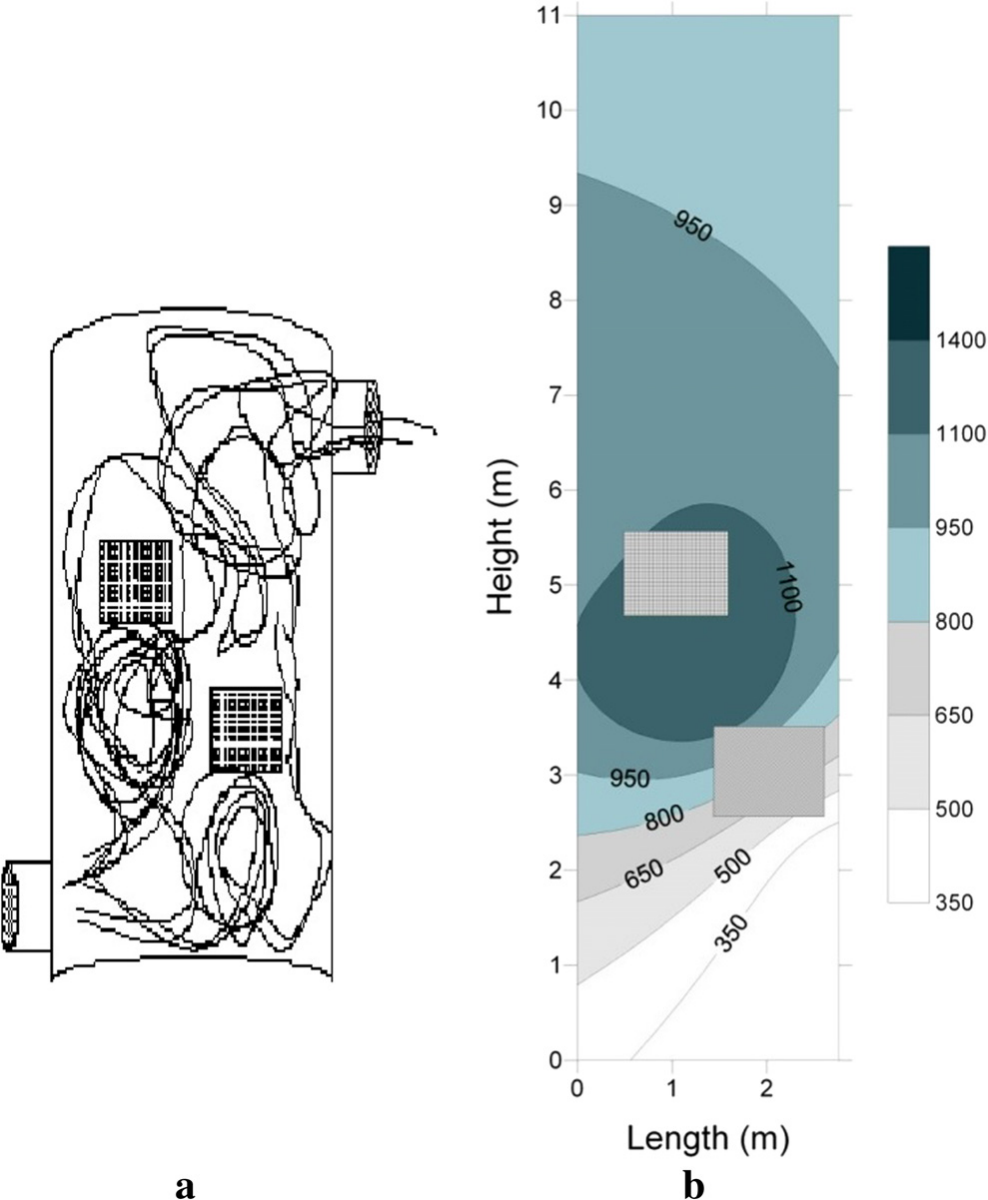
**a** The schematic motion path of gases in the incinerator for the Case 1, (**b**) The temperature profile (°C) inside the incinerator for the Case 1

Baffles were embedded in the incinerator to remove large rotating currents (or eddy). In the first case, two baffles each with an angle of 90° were installed on the height of 3 and 5 m from the bottom of the studied incinerator. Fig. 4a, shows the two-dimensional motion of particles in this case. The baffles were made of steel as the studied incinerator body. Unlike the previous case, the motion path is divided into several parts. Removal of large eddies and the sudden displacements of gaseous emissions increase the residence time and thereby optimal air mixing. The residence time calculated for this case is 3.3 s. Fig. 4b, shows the temperature profile. The maximum temperature in this case was 1400 °C. Although the heat concentration can be observed in the middle of the studied incinerator, the temperature profile is not significantly different with the previous case.

**Fig. 5:**
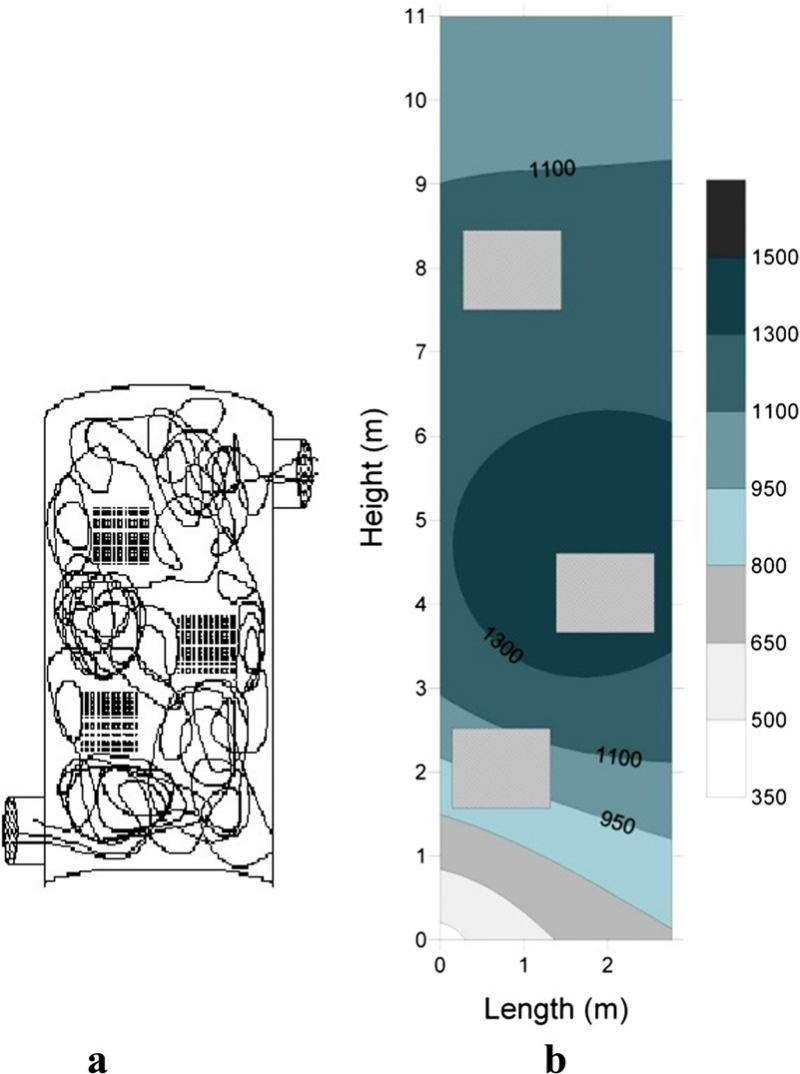
**a** Schematic motion path of gases in the incinerator for the Case 2, (**b**) The temperature profile (°C) inside the incinerator for the case 2

In the second case, 3 baffles were installed on the height of 2, 4 and 8 m from the bottom of the studied incinerator with an angle of 90 °. Fig. 5a, shows the gas motion path in this case. As shown, the large volume of the incoming gas is located adjacent to the heat source. The gas emission leaves the incinerator with a lower speed across a longer path compared to the previous case. In this case, the turbulent flow in the incinerator is stronger than the previous case. The average residence time is 3.5 s. The temperature profile is shown in Fig. 5b, Little changes in the temperature profile are observed. Like the previous case, the heat concentration is observed in the middle of the incinerator. The maximum temperature in this case is 100 °C higher than the previous case. Other studies have shown that longer path incinerator or multiple chamber incinerator can be increased PCB removal rate [15, 26].

## Conclusions

The present paper modeled a PCBs incinerator by computational fluid dynamics with the help of Fluent. First, the technical specifications of the incinerator were calculated considering the type of the pollutant. The calculated specifications were used as Fluent model inputs. Numerical modeling was performed in three different modes. In the normal mode, a cylindrical incinerator without any baffle was modeled. The results indicated the presence of vortices and cold zones in the incinerator which reduced the efficiency. Then, the impact of baffles on the heat distribution and mixing efficiency of gaseous emissions in the incinerator was studied. The baffles reduced eddies and improved the heat distribution in the incinerator. In the third case, the maximum residence time, temperature and turbulence of gaseous emissions were greater than the previous two cases. In this case, the reduced cold zones by adding baffles increased the thermal efficiency and pollutant removal by 10 % and 16 %, respectively.
